# Quantifying selective solvent transport under an electric field in mixed-solvent electrolytes†

**DOI:** 10.1039/d3sc01158e

**Published:** 2023-04-24

**Authors:** Chao Fang, David M. Halat, Aashutosh Mistry, Jeffrey A. Reimer, Nitash P. Balsara, Rui Wang

**Affiliations:** a Department of Chemical and Biomolecular Engineering, University of California Berkeley California 94720 USA ruiwang325@berkeley.edu; b Materials Sciences Division, Lawrence Berkeley National Laboratory Berkeley California 94720 USA; c Chemical Sciences and Engineering Division, Argonne National Laboratory Lemont Illinois 60439 USA

## Abstract

Electrolytes in lithium-ion batteries comprise solvent mixtures, but analysis of ion transport is always based on treating the solvents as a single-entity. We combine electrophoretic NMR (eNMR) measurements and molecular dynamics (MD) simulations to quantify electric-field-induced transport in a concentrated solution containing LiPF_6_ salt dissolved in an ethylene carbonate/ethyl methyl carbonate (EC/EMC) mixture. The selective transport of EC relative to EMC is reflected in the difference between two transference numbers, defined as the fraction of current carried by cations relative to the velocity of each solvent species. This difference arises from the preferential solvation of cations by EC and its dynamic consequences. The simulations reveal the presence of a large variety of transient solvent-containing clusters which migrate at different velocities. Rigorous averaging over different solvation environments is essential for comparing simulated and measured transference numbers. Our study emphasizes the necessity of acknowledging the presence of four species in mixed-solvent electrolytes.

## Abstract 2

Limitations of using the single-solvent approximation to quantify transport in mixed-solvent electrolytes are highlighted. A rigorous simulation approach to quantify selective solvent transport is presented and validated by electrophoretic NMR.

## Graphical abstract

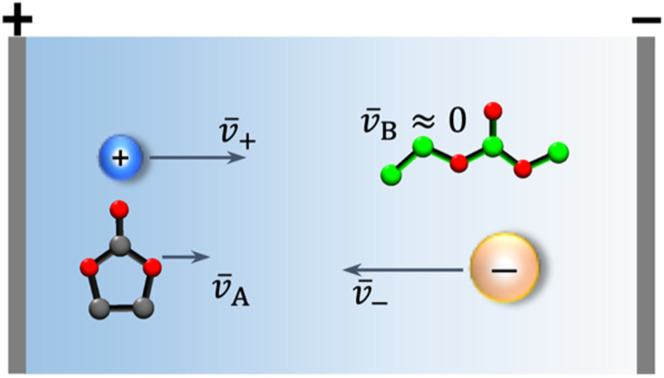

## Introduction

Many electrolytes of commercial importance consist of solvent mixtures.^1–4^ In conventional lithium-ion batteries, the solvent is a mixture of high permittivity cyclic carbonates such as ethylene carbonate (EC) and low permittivity linear carbonates such as ethyl methyl carbonate (EMC).^5^ A high permittivity component is crucial for ion dissociation but it is also characterized by high viscosities, which are detrimental for ion transport. In fact, EC is a crystalline solid at room temperature. A low permittivity component is essential for obtaining electrolyte solutions with low viscosities and enabling fast ion transport. Recent work also showed that manipulation of solvent–solvent interactions helps regulate the solvation structure and stabilize the electrolyte.^6–8^ In an important publication, Doyle and Newman modeled ion transport in lithium-ion batteries based on concentrated solution theory.^9^ While this theory is general and can be extended to include any number of components, the electrolyte was approximated as a binary electrolyte comprising two ionic species and one solvent species. Transport in binary electrolytes is governed by three Stefan–Maxwell diffusion coefficients, 𝔇_+0_, 𝔇_−0_, and 𝔇_+−_, which characterize frictional interactions between the ions (labeled + or −) and the solvent (labeled 0) and between the ions themselves.^10^ It is assumed that a mixture of low and high permittivity components can be approximately treated as a single species. Nearly every publication on modeling lithium-ion batteries is based on this assumption.

In a recent experimental publication, Wang *et al.* show that under an applied electric field, the high permittivity solvent accumulates near the negative electrode while low permittivity solvent accumulates near the positive electrode.^11^ Selective solvent transport has important implications on the overall functioning of the battery because electrochemical reaction kinetics depend on the local composition of the electrolyte solution at the electrode–electrolyte interface.^12^ Modeling solvent partitioning under an applied electric field requires going beyond the single-solvent approximation. In this work, we present the first steps toward the development of such models.

The structure of the solvation shells surrounding dissociated lithium ions in mixed-solvent electrolytes has been studied extensively by computer simulations and experiments.^13–18^ One may expect a larger number of cyclic carbonate molecules in the vicinity of lithium ions due to their high permittivity. However, other factors such as denticity of the solvent molecules and steric hindrance also play important roles.^19^ Nevertheless, it is generally accepted that solvation shells are enriched in cyclic carbonates relative to the bulk composition. This conclusion is consistent with direct spectroscopic experiments.^19–23^ However, the quantum calculation and MD simulation by Borodin *et al.* lead to the conclusion that the solvation shell is slightly enriched in linear carbonates.^24^ In an experimental study, Seo *et al.* concluded that compositions of the solvation shell and the bulk electrolyte are the same.^25^ It should be evident that even the static structure of the solvation shell in mixed-solvent electrolytes is not entirely settled. While it is reasonable to expect the local solvation shell around lithium ions to have compositions that differ from that of the bulk mixture, the effect of this local heterogeneity on continuum transport has not been established.

Quantifying transport properties in mixed-solvent electrolytes is another challenge. Continuum transport in a ternary electrolyte comprising two ionic species and two solvent species (A and B) is characterized by six Stefan–Maxwell diffusion coefficients.^10,26^ A major goal of this work is to determine these six coefficients in a mixture of LiPF_6_/EC/EMC using molecular dynamics (MD) simulations. Lithium transference in the same electrolyte was measured experimentally using electrophoretic NMR (eNMR). In binary electrolytes, cation transference is usually characterized using *t*^0^_+_, the fraction of current carried by the cation with respect to the solvent velocity.^10,27^ In our ternary electrolyte, we can therefore define two transference numbers *t*^A^_+_ and *t*^B^_+_; these transference numbers are defined with respect to the velocities of solvent A and B, respectively. EC and EMC are respectively defined as solvent A and B. We present a rigorous approach to obtain *t*^A^_+_ and *t*^B^_+_ from MD simulations and validate our approach using experimental data. The MD simulations elucidate the relationship between correlated motion of species on molecular length-scales and ion transport on continuum length-scales.

## Results and discussion

A mixture of LiPF_6_/EC/EMC, at a salt concentration of 1 M with a 1 : 1 volume ratio of EC and EMC, was used as received (“LP50” electrolyte, Sigma-Aldrich). eNMR can measure the velocities of all four species under an applied one-dimensional electric field across an electrolyte of uniform composition.^28–31^ The velocities of the cation, anion, and both solvents can be distinguished using ^7^Li, ^19^F, and ^1^H NMR measurements; the ^1^H NMR peaks of EC and EMC are well separated. The eNMR cell employed herein consists of a 5 mm NMR tube with platinum (*i.e.*, blocking) electrodes. A convection-compensated eNMR pulse sequence was employed, with bipolar electric field pulses lasting 50 ms for each polarity. The measurements were performed at a temperature of 303 K and repeated with a range of positive and negative pulsed magnetic field gradient strengths to reduce systematic errors. We found it necessary to extrapolate our measured velocities to a zero magnetic field gradient, which implies that convective effects were not fully eliminated with the pulse sequence (see Fig. S1 in the ESI†). The eNMR velocities reflect electrophoretic migration during the initial 50 ms of electric field application relative to the laboratory frame of reference.

The species velocities measured by eNMR in the laboratory frame of reference are given in Table 1. The velocities are defined as positive if they point toward the negative electrode. The measured velocities of the cation are positive, while those of the anion are negative. The velocities of EC and EMC are much smaller in magnitude. It is important to account for the motion of electrodes, especially when species velocities are small. In ref. 32, we present a method to determine the velocity of the electrodes in an eNMR experiment. Applying this methodology to the present data set, the obtained species velocities could be converted into the electrode reference frame; see Section I in the ESI† for details. These values are also listed in Table 1. In the electrode reference frame, EC has a positive velocity while the EMC velocity is negligible. In the discussions below, we only use velocities measured in the electrode reference frame.

**Table 1:** eNMR velocities of ions and solvent molecules under an applied electric field. All velocities reflect an applied electric field of 1 V mm^−1^; measurements were performed in a range of electric fields and velocities were scaled to 1 V mm^−1^. Velocities were converted to the moving electrode reference frame using the method of ref. 32, described further in the ESI

Species	Velocity in the laboratory frame (μm s^−1^)	Velocity in the moving electrode frame (μm s^−1^)
Cation (+)	3.7 ± 0.3	4.2 ± 0.4
Anion (−)	−7.2 ± 0.2	−6.8 ± 0.3
EC (A)	0.3 ± 0.2	0.8 ± 0.3
EMC (B)	−0.5 ± 0.2	0.0 ± 0.3

The transference number is defined as:^33^1a
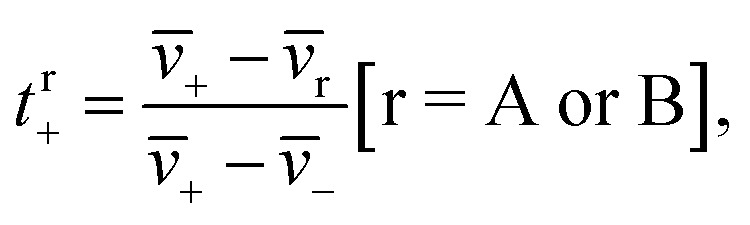
where *v̄*_*i*_ is the average velocity of species *i* (*i* = +, −, A (EC), or B (EMC)), *v̄*_r_ is the reference velocity that can be chosen to be *v̄*_A_ or *v̄*_B_, and *t*^r^_+_ is the corresponding cation transference number with respect to *v̄*_r_. We use the symbol *v̄*_*i*_ in eqn (1a) to acknowledge that each species can be in a variety of solvation environments and the measured eNMR velocities represent averages over them.

We can define the transference number with respect to the mass average velocity of the solvents,

1b *v̄*_0_ = (*ω*_A_*v̄*_A_ + *ω*_B_*v̄*_B_)/(*ω*_A_ + *ω*_B_),

where *ω*_*i*_ is the mass fraction of species *i*. If we approximate our ternary electrolyte to be a binary electrolyte with a single-solvent species, then the transference number with respect to *v̄*_0_ is given by1c
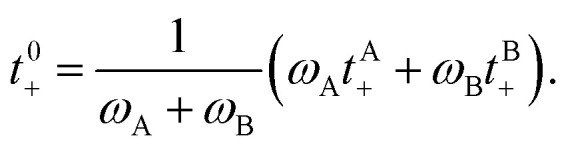
In all previous studies, the cation transference number has been determined using the concentrated solution theory for a binary electrolyte.^10,27,34^ The transference number reported in these studies corresponds to *t*^0^_+_. Another common choice for the reference velocity is the mass averaged velocity of all species including the ions (*v̄*_M_). The transference number with respect to *v̄*_M_ is given by^10,35^

1d *t*^M^_+_ = *ω*_A_*t*^A^_+_ + *ω*_B_*t*^B^_+_ + *ω*_−_.

**Fig. 1:**
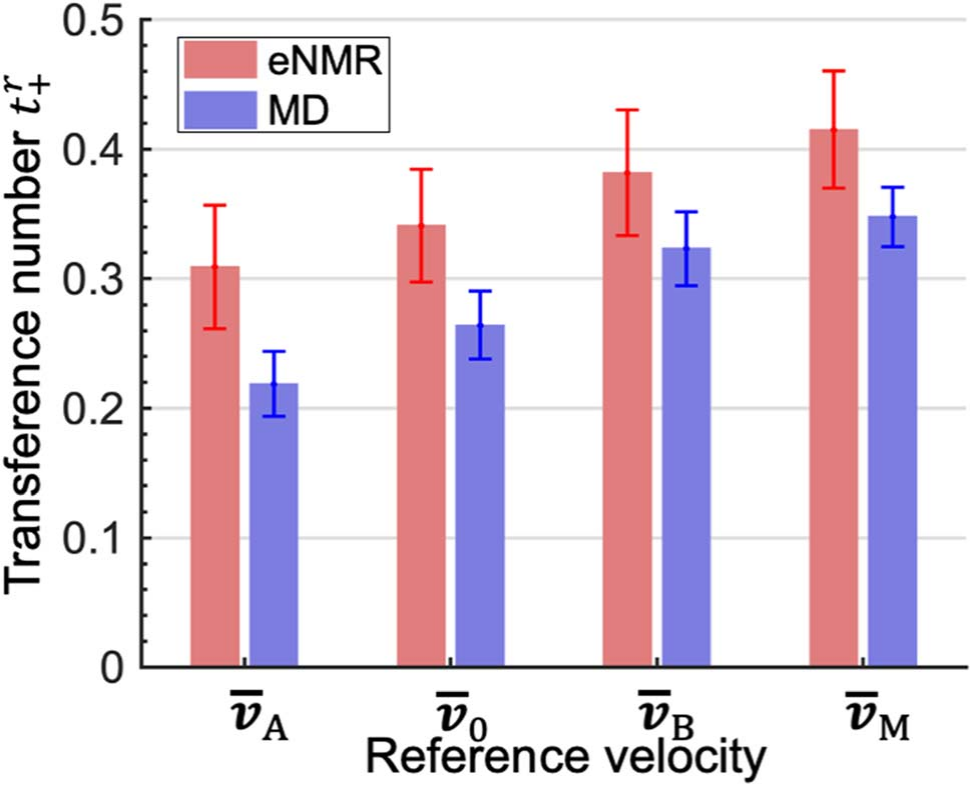
Cation transference numbers *t*^r^_+_ with respect to different reference velocities: *v̄*_A_, *v̄*_0_, *v̄*_B_, and *v̄*_M_. r represents different reference velocities. The subscripts A, B, 0, and M correspond to EC, EMC, the mass average velocity of the solvents and the center of mass of all species, respectively.

The experimentally measured transference numbers defined using eqn (1a), (1c) and (1d) are shown in Fig. 1. Our next objective is to compare these measurements with predictions based on computer simulations.

MD simulations were performed on the same LiPF_6_/EC/EMC system studied experimentally (1 M concentration with a 1 : 1 solvent volume ratio). The ions and solvent molecules were modeled based on the widely used all-atom optimized potentials for liquid simulations (OPLS-AA) force field.^36,37^ While the available Lennard-Jones potential, partial atomic charges, and bonded interactions were directly applied to Li^+^ and PF_6_^−^ ions, the partial atomic charges for EC and EMC were separately fitted using the RESP method^38,39^*via* the Gaussian package^40^ and Antechamber package.^41^ Equilibrium *NpT* simulations were conducted at 303 K and 1 bar using the Gromacs package (version 5.1.4).^42^ Each simulation was run for 200 ns. Four independent simulations were performed to enable better sampling of transport coefficients. Further details are provided in Section II in the ESI.†

To rigorously quantify the correlated motion of the four species *via* simulation, we employ the transport framework proposed by Wheeler and Newman.^43,44^ This system is characterized by six transport coefficients and they are defined with respect to a reference species. If the reference species is A (EC), then the transport coefficients are given by2a
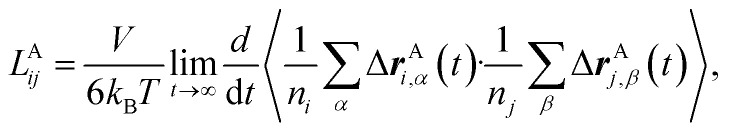
where *i* and *j* denote the non-reference species (B, +, or −), *V* is the system volume, *k*_B_*T* is the thermal energy, *t* is the time, *n*_*i*_ is the number of molecules or ions of species *i*, and Δ***r***^A^_*i*,*α*_(*t*) = ***r***^A^_*i*,*α*_(*t*) − ***r***^A^_*i*,*α*_(0) is the displacement vector of the *α*^th^ molecule or ion of species *i*, where ***r***^A^_*i*,*α*_(*t*) is the position with respect to the average position of species A at time *t*. Each *L*^A^_*ij*_ can be evaluated from the simulation trajectories. *L*^A^_*ij*_s give a transference number with respect to the species velocity of A as:2b
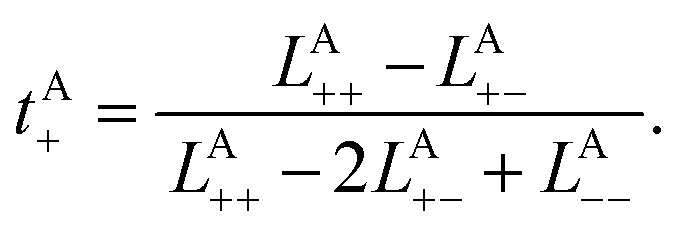


The results of the simulations can be compared with experimentally measured *t*^A^_+_ using eqn (1a).

Likewise, the cation transference number with respect to the species velocity of B (EMC), *t*^B^_+_, can be obtained from3a
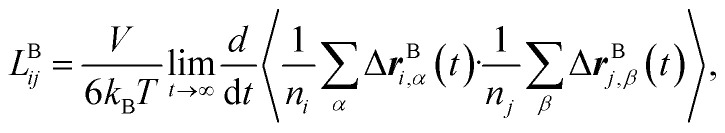
where *L*^B^_*ij*_s are the transport coefficients with respect to species B. *L*^B^_*ij*_s give a transference number with respect to the species velocity of B as follows:3b
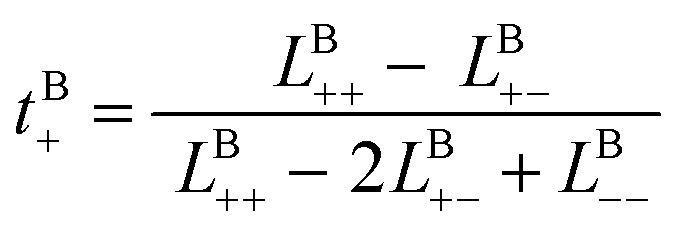


The results of the simulations can also be compared with experimentally measured *t*^B^_+_ using eqn (1a).

The application of *L*^A^_*ij*_s is equivalent to using six Stefan–Maxwell diffusion coefficients. The computed values of *L*^A^_*ij*_ can be used to determine the six Stefan–Maxwell diffusion coefficients, 𝔇_*ij*_ (*i*,*j* = *A*, *B*, +, or −, and *i* ≠ *j*),^10^ using the Onsager transport equation. The average species velocities are related to *L*^A^_*ij*_ by4a
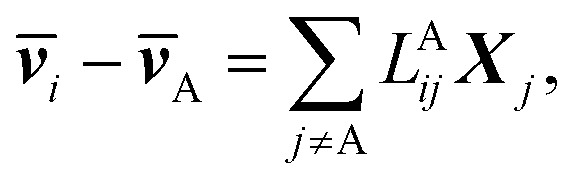
where ***v̄***_*i*_ is the average velocity of species *i* (*i* ≠ *A*) and ***X***_*i*_ is the driving force for the diffusion of species *i*, which is related to the concentration *c*_*i*_ and the chemical potential *μ*_*i*_ by ***X***_*i*_ = −*c*_*i*_∇*μ*_*i*_. Note that the species velocities in eqn (4a) are in the general vector form, which is different from the scalar eNMR velocities used in eqn (1). Due to the Onsager reciprocal relation,^45^*L*^A^_*ij*_ = *L*^A^_*ji*_. Matrix **L**^A^ is symmetric with each row or column representing species of B, +, or −.

One can re-express eqn (4a) as the Stefan–Maxwell equation:4b
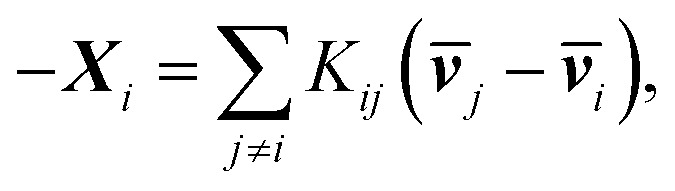
where *K*_*ij*_ is the friction coefficient that is related to 𝔇_ij_, *K_ij_* = *RTc_i_c_j_*/(*c*_t_𝔇*_ij_*), in which *R* is the gas constant, *c*_*i*_ is the molar concentration of species *i*, and *c*_t_ is the total molar concentration in the entire system. **K** is a 4 by 4 matrix with undefined diagonal elements. The Stefan–Maxwell equation can be rewritten as:4c
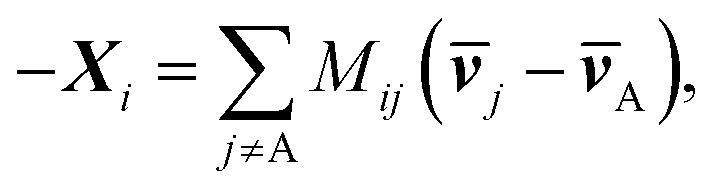
where *M*_*ij*_ is defined as 
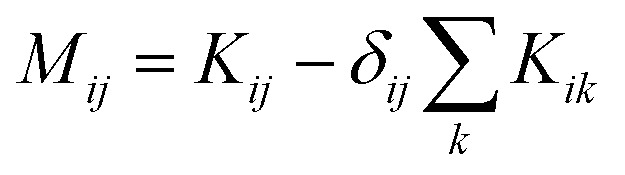
. **M** is a 4 by 4 matrix. **M** and **L**^A^ are related by **M**^A^ = −(**L**^A^)^−1^, where **M**^A^ is obtained by removing the row and column of **M** corresponding to species A. The remaining elements of **M** are completed by the definition of *M*_*ij*_ as: 
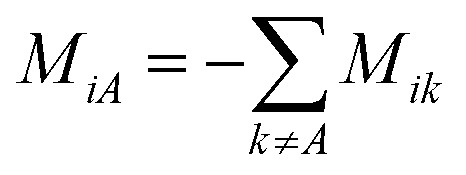
. Finally, each 𝔇_ij_ corresponds to one nondiagonal element of **M**: 𝔇_ij_ = *RTc_i_c_j_*/(*c_t_M_ij_*). Note that 𝔇_ij_s are independent of the reference species. While we have presented the approach based on *L*^A^_*ij*_, one could also use *L*^B^_*ij*_ to arrive at the same set of 𝔇_ij_s.

**Fig. 2:**
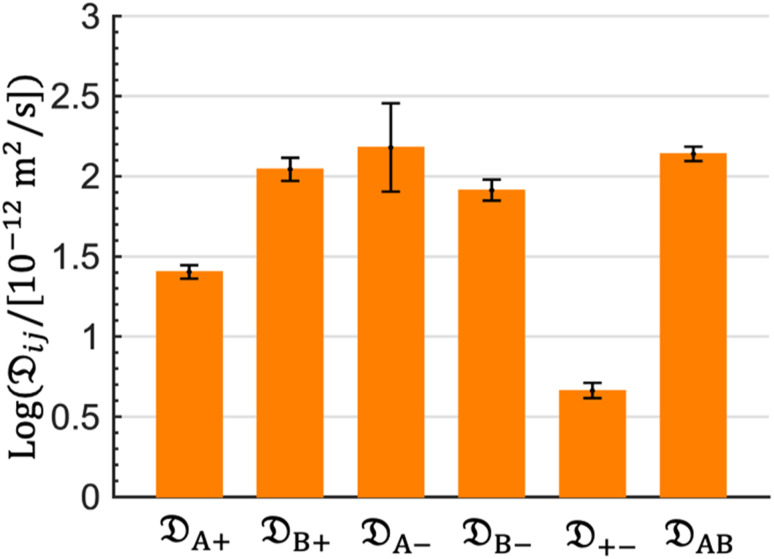
Stefan–Maxwell diffusion coefficients calculated from MD simulations. The diffusivities are normalized by a value of 10^−12^ m^2^ s^−1^ and are shown on logarithm base 10 scale. A and B represent EC and EMC species, respectively.

The computed Stefan–Maxwell diffusivities are shown in Fig. 2. The smallest Stefan–Maxwell diffusivity is 𝔇_+−_, indicating that the dominant frictional interaction is between the cation and the anion. 𝔇_A+_ is lower than 𝔇_B+_ by a factor of 4.4. This implies that the frictional interaction between the cation and solvent A (EC) is much larger than that between the cation and solvent B (EMC). This provides a qualitative explanation of our observation that *v̄*_A_ > *v̄*_B_ (Table 1). 𝔇_B+_, 𝔇_A−_, 𝔇_B−_, and 𝔇_AB_ are similar in magnitude, implying that frictional interaction between the cation and solvent B is similar to those between the anion and solvent A, the anion and solvent B, and solvents A and B.

The *L*^A^_*ij*_ and *L*^B^_*ij*_ are used to calculate *t*^A^_+_ and *t*^B^_+_ using eqn (2b) and (3b). In addition, we calculate *t*^0^_+_ and *t*^M^_+_ using eqn (1c) and (1d). We compare the four theoretically calculated transference numbers with the measured values in Fig. 1. Both eNMR measurements and MD simulations indicate that *t*^A^_+_ < *t*^0^_+_ < *t*^B^_+_ < *t*^M^_+_. The fact that *t*^A^_+_ is less than *t*^B^_+_ implies that the two solvents cannot be treated as a single entity. The cation selectively drags more EC molecules than EMC molecules as it migrates under an electric field. The difference between the two transference numbers is not very large; the theoretical ratio of *t*^B^_+_/*t*^A^_+_ is 1.48 ± 0.21. In addition, *t*^0^_+_ is an intermediate between *t*^A^_+_ and *t*^B^_+_ because we have taken *v̄*_0_ to be the mass averaged velocity of EC and EMC, as stated in eqn (1b). *t*^M^_+_ is larger than *t*^0^_+_ because they are related by *t*^M^_+_ − *t*^0^_+_ = (1 − *t*^0^_+_)*ω*_−_ − *t*^0^_+_*ω*_+_. The right side of this equation is positive because *ω*_−_ is significantly larger than *ω*_+_.

**Fig. 3:**
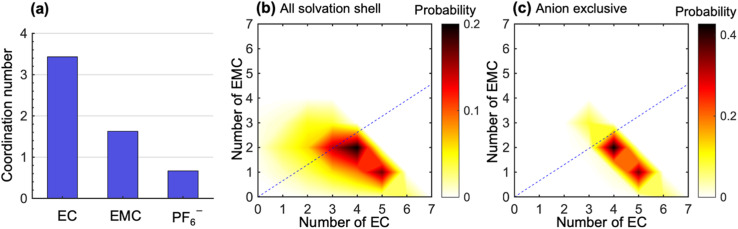
Static ion solvation structures. (a) Coordination numbers of EC, EMC, and PF_6_^−^. Probability distribution of the number of EC and EMC molecules within (b) all Li^+^ solvation shells and (c) solvation shells that are exclusive of anions. The dashed lines in (b) and (c) denote a specific composition of the two solvent species, in which the ratio between EC and EMC molecules is the same as that in the bulk electrolyte. The color bars in (b) and (c) denote the probability of obtaining a specific solvation structure in the contour plots.

To study the molecular underpinnings of cation transference, we examine Li^+^ solvation structures obtained from simulations. Fig. 3a compares the average coordination number of solvent molecules and anions within each solvation shell of Li^+^. The coordination number is calculated from the pair correlation function between Li^+^ and the carbonyl oxygen atoms on the solvent molecules or the phosphorus atoms on PF_6_^−^ (see Fig. S7 in the ESI†). The EC coordination number is two times higher than the EMC coordination number. However, a nonnegligible amount of PF_6_^−^ ions is also present in the solvation shell. The coordination number indicates a preferential solvation of EC over EMC. This is consistent with many previous simulations using different force fields.^15–18^ In addition, the ratio between the fraction of solvated EC and that of EMC (1.38 in our simulation) is in quantitative agreement with the solvation power series of common lithium electrolytes determined by experiments at similar salt concentrations.^19^

Fig. 3b shows the distribution of the solvation structures that underlie the averages for EC and EMC reported in Fig. 3a. This figure shows two-dimensional probability distributions of the number of EC and EMC molecules within the solvation shell. We focus on the solvation of Li^+^;^46,47^ the radial distribution functions of Li^+^ to solvents in LiPF_6_/EC/EMC mixtures have much more pronounced features than those of the anion to solvents (see Fig. S8 in the ESI†). In Fig. 3b, we see two strong peaks corresponding to Li^+^ solvation shells with (5EC + 1EMC) and (4EC + 2EMC). The two peaks are smeared and tilted toward the bottom left, which indicates fewer solvent molecules in the solvation shell due to the presence of anions. These shells have small contribution to the overall migration of Li^+^. Therefore, we examine the solvation shells that exclude anions in Fig. 3c. The fraction of these solvation shells, which will be strongly coupled to the migration of Li^+^, is 0.47. Li^+^ in these environments are primarily solvated by (5EC + 1EMC) and (4EC + 2EMC), indicating a more substantial solvation provided by EC molecules in anion-exclusive shells. The EC : EMC ratio in the bulk electrolyte is denoted by the dashed lines in Fig. 3b and c. It is evident that the shells surrounding dissociated Li^+^ ions are significantly enriched in EC by a factor as high as 3 compared to the bulk. Based on this observation, one might expect that cation transference numbers with respect to velocities of EC and EMC to be very different. However, Fig. 1 shows that this is not the case. Further dynamic analysis is thus needed to elucidate the relationship between local solvation and cation transference.

**Fig. 4:**
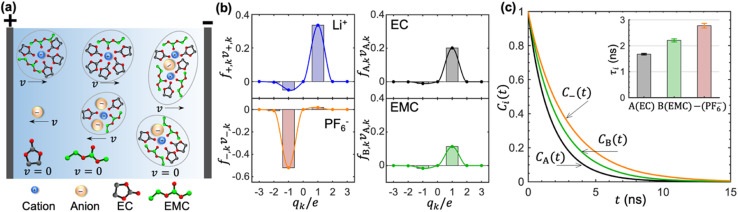
Dynamic picture of the motion of species under an applied electric field. (a) A schematic of the velocities of clusters containing different numbers of solvent molecules and ions under an electric field. (b) Estimation of the electric-field-induced drift of Li^+^, PF_6_^−^, EC, and EMC in cluster types carrying a net charge of *q*_*k*_. The velocities are normalized by the absolute value of a single anion under the same electric field. (c) Residence time auto-correlation functions between the cation and the solvent molecules or the anion. The inset shows the mean residence times.

In concentrated electrolytes, the cations are present in various types of transient clusters that contain different net charges and different numbers of solvent molecules. To enable a quantitative estimation of the migration of the species in these clusters, we adopt the cluster approximation approach developed in ref. 48. In this approach, clusters of species containing different numbers of cations, anions, and solvent molecules are identified using the single-linkage algorithm. The interactions between clusters are ignored. In ref. 49, this approach has been extended to calculate the migration velocity of different clusters under an applied electric field. This extension provides molecular insights into the underpinnings of cation transference. Ion-containing clusters are constructed from the linkage between individual cations and anions. The linkage is successfully built when the anion is within the solvation shell of Li^+^, which is determined based on the distance between the closest fluorine atom and Li^+^. Similarly, the linkage between a cation and a solvent molecule is successfully built when the carbonyl oxygen atom is within the solvation shell of Li^+^. Schematics of clusters thus obtained are shown in Fig. 4a. We also show isolated EC, EMC, and PF_6_^−^ in Fig. 4a.

Each of the clusters and free species will migrate with velocities that depend on both net charge and size as illustrated in Fig. 4a. The velocities are defined as positive if they point toward the negative electrode and are negative otherwise. The clusters are grouped based on their net charge *q*_*k*_ to facilitate the analysis of the contribution to the species velocity from different clusters. The average velocity of species *i* due to a particular cluster type with charge *q*_*k*_ will depend on the fraction of species in that cluster type *f*_*i*,*k*_ and its velocity *v*_*i*,*k*_:5a
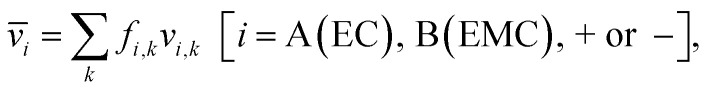
where *v*_*i*,*k*_ is based on the electrical mobility that is determined using net-charge and diffusivity based on the Stokes–Einstein equation (see Section V in the ESI†).

Fig. 4b shows the distribution of *f*_*i*,*k*_*v*_*i*,*k*_ for all four species. The cation velocity is mainly due to clusters with *q*_*k*_ = +1*e* and −1*e*. Included in this figure are contributions from other cluster types, but their contributions are negligible. The anion velocity is dominated by clusters with *q*_*k*_ = −1*e* with a relatively small contribution from clusters with *q*_*k*_ = +1*e*. The difference between cation and anion velocities arises from the fact that the electrical mobility of anion-containing positive clusters is smaller than that of cation-containing negative clusters. Both solvent velocities are dominated by clusters with *q*_*k*_ = +1*e*. However, the EC peak is higher than the EMC peak, indicating that EC molecules are selectively dragged by the motion of clustered cations.

The species velocities can be used to estimate transference numbers based on the cluster approximation as follows:5b
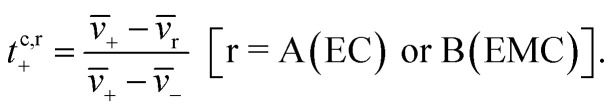
The velocities used to calculate *t*^c,A^_+_ and *t*^c,B^_+_ in eqn (5b) are based on eqn (5a). *t*^c,A^_+_ and *t*^c,B^_+_ thus obtained are 0.11 ± 0.005 and 0.23 ± 0.002, respectively. The qualitative trend obtained using the cluster approximation is consistent with both eNMR velocities and the rigorous analysis of simulation data. Quantitatively, however, cluster approximation gives transference numbers that are lower than the experimental values by a factor of two. The ratio of the transference numbers obtained using the cluster approximation (*t*^c,B^_+_/*t*^c,A^_+_ = 2.05 ± 0.09) is larger than those obtained from the full calculation (*t*^B^_+_/*t*^A^_+_ = 1.48 ± 0.21) and eNMR experiments (*t*^B^_+_/*t*^A^_+_ = 1.24 ± 0.25). These quantitative deviations occur because the cluster approach only accounts for static distributions, whereas the finite lifetime of clusters is not taken into account.

The timescales of cation–solvent and cation–anion associations are evaluated by introducing the residence time autocorrelation function as:^50^6
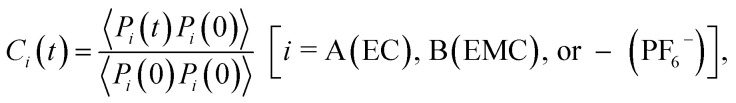
where *P*_*i*_(*t*) equals 1 when an individual solvent molecule or anion of species *i* coordinates a Li^+^ from time 0 to time *t* and equals 0 otherwise. Fig. 4c shows the decay of *C*_A_(*t*), *C*_B_(*t*), and *C*_−_(*t*) as a function of time. The average residence times of all three species are between 1 and 5 ns. The three *C*_*i*_(*t*)s are fitted using a stretched exponential function (exp[(−*t*/*τ*_*i*_)^*β*^]) to extract the mean residence time *τ*. The inset of Fig. 4c shows these results *τ*_A_ < *τ*_B_ < *τ*_−_. The surprising result is that *τ*_B_ is larger than *τ*_A_, implying that EMC molecules tend to be dragged by Li^+^ for a slightly longer time when compared to EC molecules. This result explains why *t*^c,A^_+_ and *t*^c,B^_+_ are lower than *t*^A^_+_ and *t*^B^_+_, and *t*^c,B^_+_/*t*^c,A^_+_ > *t*^B^_+_/*t*^A^_+_. The effect of the finite lifetimes of clusters is captured in the full calculations but not in the cluster approximation. The cluster approximation assumes that the lifetime of all clusters is infinite. It is nevertheless valuable as it provides insight into the dominant solvation structures and their impact on cation transference.

## Conclusions

In summary, we have combined eNMR experiments and MD simulations to quantify transport in a 1 mol L^−1^ solution of LiPF_6_ dissolved in an equal volume mixture of EC (A) and EMC (B). All electrolytes used in lithium-ion batteries comprise mixtures of solvents. Our goal is to quantify the selective transport of one of the solvents due to differences in the coupling between ions and solvent molecules. Selective transport is characterized by defining two transference numbers, *t*^A^_+_ and *t*^B^_+_. These transference numbers reflect the fraction of current carried by the cation relative to the velocities of the two different solvent species. The eNMR experiments indicated that *t*^B^_+_/*t*^A^_+_ = 1.24 ± 0.25. The Stefan–Maxwell diffusivities are evaluated from MD simulations and the calculated ratio *t*^B^_+_/*t*^A^_+_ is 1.48 ± 0.21. The disparity between *t*^A^_+_ and *t*^B^_+_ indicates that EC (A) is selectively transported to the negative electrode. This result is consistent with the experimental data in ref. 11. The cluster approximation is used to provide molecular-level insights into the factors that affect cation transference. Our concentrated electrolyte contains a variety of clusters with different dynamic features that must be averaged to obtain *t*^A^_+_ and *t*^B^_+_, parameters that reflect transport on the continuum scale. The methodology of combining eNMR experiments and MD simulations to quantify selective solvent transport can be applied to other mixed-solvent electrolytes containing different salts and solvents provided they contain NMR-active nuclei. Our study highlights the limitations of using the single-solvent approximation to quantify transport in electrolytes comprising two solvent species. Full characterization of such systems will require the determination of six Stefan–Maxwell diffusivities, 𝔇_ij_s. Our work, which focuses on *t*^A^_+_ and *t*^B^_+_, represents the first step toward full characterization.

## Data availability

The ESI† contains the eNMR and simulation details. Furthere data are available from the corresponding authors upon request.

## Author contributions

This project was conceived by R. W. and N. P. B. The computational studies were performed and analyzed by C. F. eNMR experiments were performed and analyzed by D. M. H. The manuscript was written by C. F., D. M. H., N. P. B., and R. W. All the authors approved the final version of the manuscript.

## Conflicts of interest

There are no conflicts to declare.

## Supplementary Material

SC-014-D3SC01158E-s001
