# Supplementary material for: Quantifying selective solvent transport under an electric field in mixed-solvent electrolytes
Source: Chem Sci. 2023 Apr 24;14(20):5332–9. doi: 10.1039/d3sc01158e (PMC10207890; doi:10.1039/d3sc01158e)
Supplement: SC-014-D3SC01158E-s001 [file SC-014-D3SC01158E-s001.pdf]

Electronic Supplementary Material (ESI) for Chemical Science.
This journal is © The Royal Society of Chemistry 2023

Electronic Supplementary Information for

Quantifying Selective Solvent Transport under an Electric Field in Mixed-Solvent Electrolytes

Chao Fang,[1,2†] David M. Halat,[1,2†] Aashutosh Mistry,[3] Jeffrey A. Reimer,[1,2] Nitash P. Balsara,[1,2] and Rui Wang[1,2*]

[1]Department of Chemical and Biomolecular Engineering, University of California, Berkeley, California 94720, United States of America

[2]Materials Sciences Division, Lawrence Berkeley National Laboratory, Berkeley, California 94720, United States of America

[3]Chemical Sciences and Engineering Division, Argonne National Laboratory, Lemont, Illinois 60439, United States of America

[†] Authors contribute equally

[*] Corresponding author: ruiwang325@berkeley.edu

I. eNMR experiments

All ^{7}Li, ^{19}F, and ^{1}H NMR experiments were performed at a field strength of 16.4 T using a 700 MHz Bruker Avance I spectrometer; a Bruker 5 mm double-resonance broadband observe (BBO) probe (for ^{7}Li and ^{1}H measurements) or a Bruker 5 mm triple-resonance inverse (TXI) probe (for ^{19}F measurements) were used. Both BBO and TXI probes were equipped with a *z*-axis gradient coil with a maximum pulsed field gradient (PFG) strength of ~0.5 T/m and variable-temperature control. Distinct ^{1}H resonances corresponding to the EC and EMC components of the electrolyte could be resolved, allowing for the independent measurement of velocities of both species; the ^{7}Li and ^{19}F resonances unambiguously corresponded to Li^+ cations and $TFSI^-$ anions, respectively. The eNMR instrumentation employed in this work is based upon the setup described previously,[1] and was supplied by P&L Scientific Instrument Service (www.plscientific.se; Lidingö, Sweden); details of our eNMR measurements have been previously reported in detail.[2] The electrolyte sample was loaded into the eNMR cell within an argon-filled glovebox. We used a convection-compensated double stimulated echo (DSTE) PFG-NMR sequence,[3] with electric field pulses of opposite polarity applied during the two halves of the sequence.[4-6] Typical applied voltages ranged from 5–15 V. Although a range of electric fields were applied, in this work all eNMR species velocities are reported relative to an applied electric field of 1 V/mm. For all experiments, the drift time Δ during which the electric field was applied was fixed at 100 ms. The recycle delay was set to 120 s, to allow for equilibration following the electric field pulses. The calibrated sample temperature was 30°C for all measurements. Calibration of the electric field was previously performed with a 10 mM solution of tetramethylammonium bromide (TMABr) in D_2O (supplied by P&L Scientific) at 25°C. Analysis of eNMR phase shifts was performed as previously described[2] using an automated procedure comparing the "phase spectra" of the eNMR data**.** Dependence of the extracted velocities on the applied magnetic field strength parameters was observed (Fig. S1), which likely reflects a distribution of velocities coupled with a distribution of self-diffusion coefficients; to obtain average species velocities that were not weighted by diffusivities, extrapolation of the velocity data to zero applied magnetic field was performed.

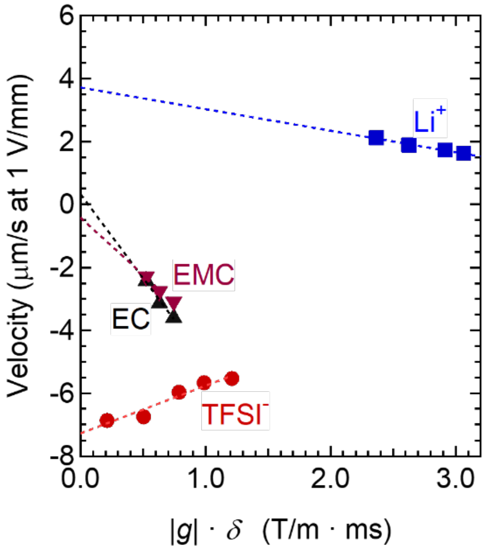

Figure S1. Species velocities measured by electrophoretic NMR in the laboratory frame of reference, as a function of the magnitude of the product of the applied magnetic field gradient strength, $|g|$, and the applied magnetic field gradient length, δ. Velocities were measured at multiple electric field gradient strengths and scaled to 1 V/mm. Measurements reflect an average of positive and negative applied magnetic field gradient strengths. Linear extrapolation of the species velocities is depicted with dashed lines.

Conversion of the eNMR species velocities from the laboratory frame of reference to the moving electrode reference frame requires knowledge of the conductivity and partial molar volume of the electrolyte.[7] Experimental conductivity data on $LiPF_6$:EC:EMC electrolytes were previously reported by Wang *et al.*[8] We interpolated this data, acquired at 30°C, to obtain the conductivity of the composition studied in this work (1 mol/L $LiPF_6$; 1:1 volume ratio, EC:EMC), as shown in Fig. S2. Wang *et al.*[8] also reported concentration-dependent density data for $LiPF_6$:EC:EMC electrolytes, which can be converted to partial molar volumes using

$$\bar{V}_e = \left(M - \frac{\mathrm{d}\rho}{\mathrm{d}c}\right) / \left(\rho - c\frac{\mathrm{d}\rho}{\mathrm{d}c}\right) \quad \text{(S1)}$$

where $\bar{V}_e$ is the partial molar volume of salt in the electrolyte, M is the molar mass of the salt, ρ is the density of the electrolyte, and c is the molar concentration. We interpolated the previously reported density data acquired at 30°C to obtain the density as a function of concentration at the desired composition (1:1 volume ratio, EC:EMC), as shown in Fig. S3a. Using Eq. S1, $\bar{V}_e$ was calculated and found to be 0.0558 L/mol at a concentration of 1 mol/L $LiPF_6$ (Fig. S3b).

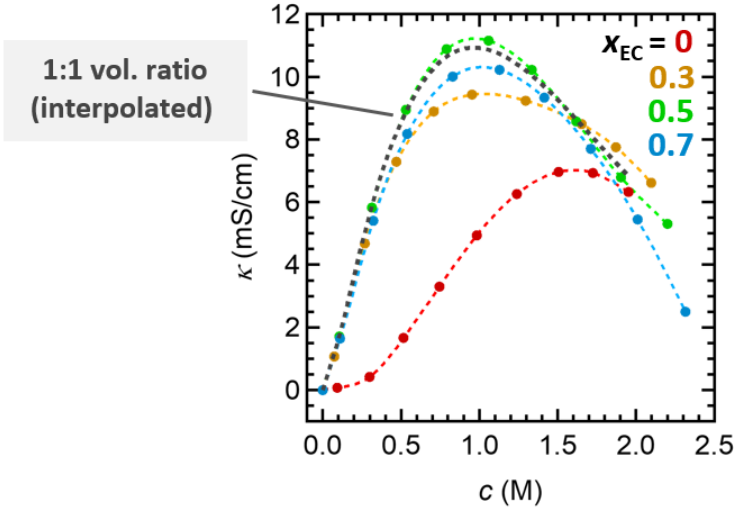

Figure S2. Experimental conductivity measurements reported by Wang *et al.*[8] on a series of electrolytes comprising $LiPF_6$ as a function of salt concentration dissolved in EC:EMC, at various solvent compositions. The colored lines depict weight ratios x_{EC} = 0, 0.3, 0.5, and 0.7. The dashed grey line depicts the interpolated conductivity corresponding to electrolytes containing an equal volume mixture of EC and EMC (*i.e.,* x_{EC} = 0.44). The interpolated conductivity of a 1 mol/L solution of the equal volume mixture is 10.9 mS/cm.

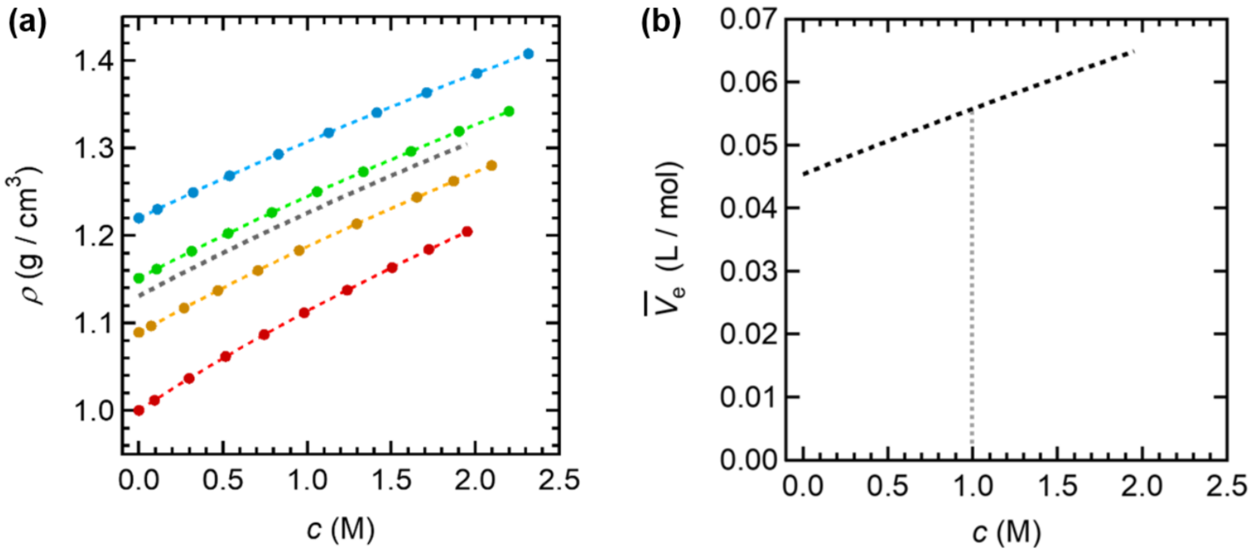

Figure S3. **(a)** Experimental density measurements reported by Wang *et al.*[8] on a series of electrolytes comprising $LiPF_6$ as a function of salt concentration dissolved in EC:EMC, at various solvent compositions. The colored lines depict weight ratios x_{EC} = 0, 0.3, 0.5, and 0.7. The dashed grey line depicts the interpolated density corresponding to an equal volume mixture of EC and EMC (i.e., x_{EC} = 0.44). **(b)** Partial molar volume of the electrolyte, $\overline{V}_e$, calculated from the density data according to Eq. S1, as a function of salt concentration. The electrolyte partial molar volume of a 1 mol/L solution of the equal volume mixture (dotted vertical line) is 0.0558 L/mol.

Using MD simulations described in the following sections, we also obtained simulated densities for electrolytes comprising a 1:1 volume ratio of EC:EMC as a function of salt concentration. Using Eq. S1, $\bar{V}_e$ was calculated and found to be 0.0532 L/mol at a concentration of 1 mol/L $LiPF_6$ (Fig. S4) representing excellent agreement with the experimental data of Wang *et al.*[8]

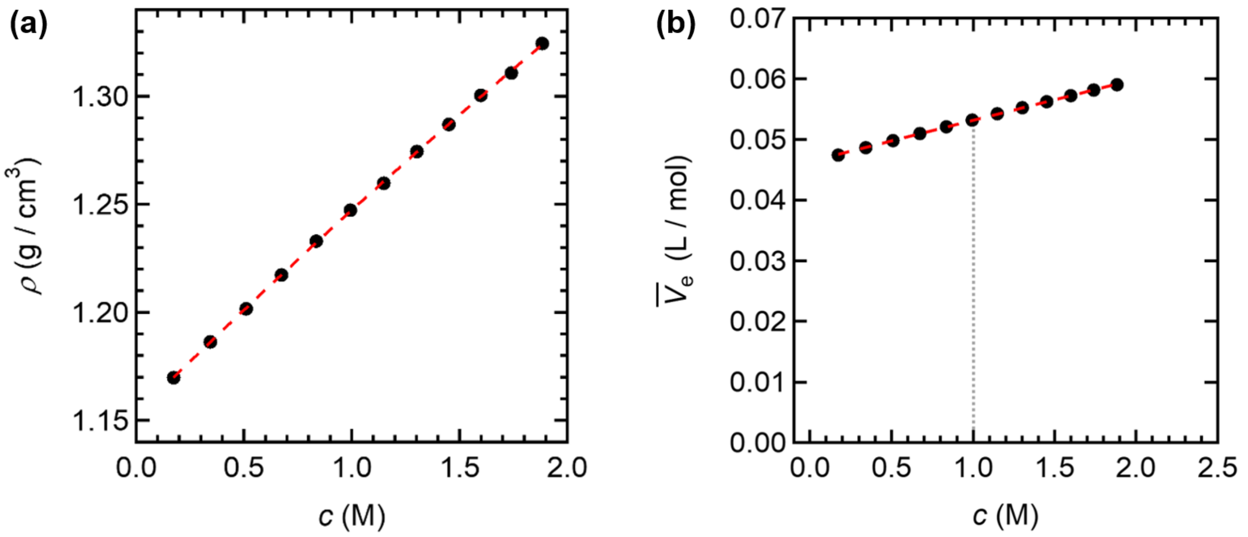

Figure S4. **(a)** Density simulated by MD in the present work, on a series of electrolytes comprising $LiPF_6$ as a function of salt concentration dissolved in an equal volume mixture of EC and EMC. **(b)** Partial molar volume of the electrolyte, $\bar{V}_e$, calculated from the simulated density data according to Eq. S1, as a function of salt concentration. The electrolyte partial molar volume of a 1 mol/L solution of the equal volume mixture (dotted vertical line) is 0.0532 L/mol.

Reference frame conversion was carried out by calculating the velocity of the electrode–electrolyte interface, $v_{\text{interface}}$, in the laboratory frame of reference, using the equations previously derived in Halat *et al.*[7], *i.e.*,

$$v_{\text{interface}} = (c\bar{V}_e)\bar{v}_+ + (1 - c\bar{V}_e)\bar{v}_0 - \frac{\bar{V}_e}{\nu_+ z_+ F}\kappa\frac{V}{L} \qquad \text{(S2)}$$

or

$$v_{\text{interface}} = (c\bar{V}_e)\bar{v}_- + (1 - c\bar{V}_e)\bar{v}_0\,, \qquad \text{(S3)}$$

where $\bar{v}_+$, $\bar{v}_-$ and $\bar{v}_0$ are the measured eNMR species velocities of the cation, anion, and solvent, ν_+ is the number of moles of cations into which a mole of salt dissociates (equal to 1 for a univalent salt), z_+ is the cationic charge (equal to +1), κ is the conductivity, and V/L is the electric field, taken to be 1 V/mm as the reported species velocities have been normalized to this value. In using

Eqs. S2 and S3, we assumed $\bar{v}_0$ to be the simple average of the measured velocities of the EC and EMC species. The experimental conductivity and partial molar volume were taken from Figs. S2 and S3; using the simulated partial molar volume (Fig. S4) had no effect on the calculated values within error. Table S1 shows the calculated values of $v_{\text{interface}}$, and the eNMR species velocities adjusted by this value, *i.e.*, in the moving electrode reference frame. Within error, no difference was observed in the values calculated using Eq. S2 or Eq. S3.

	$v_{\text{interface}}$ (μm/s)	$\bar{v}_+$ (μm/s)	$\bar{v}_-$ (μm/s)	$\bar{v}_{EC}$ (μm/s)	$\bar{v}_{EMC}$ (μm/s)
Eq. S2	-0.50 ± 0.05	4.2 ± 0.4	-6.8 ± 0.3	0.8 ± 0.3	0.0 ± 0.3
Eq. S3	-0.51 ± 0.05	4.2 ± 0.4	-6.8 ± 0.3	0.8 ± 0.3	0.0 ± 0.3

Table S1. Calculated values of electrode–electrolyte interface, $v_{\text{interface}}$, from equations Eq. S2 and Eq. S3, using the experimental conductivity and partial molar volume data depicted in Fig. S2 and S3, respectively. Species velocities converted to the moving electrode reference frame (*i.e*, adjusted by the calculated values of $v_{\text{interface}}$) are also tabulated.

II. MD simulations

MD simulations were performed using the Gromacs package (version 5.1.4).[9] The bonds involving hydrogen atoms are constrained using the LINCS algorithm.[10] An *NpT* ensemble with velocity-rescale thermostat[11] and Berendsen barostat[12] was implemented at 303 K and 1 bar, respectively. A global cutoff of 1.2 nm is used for computing Lennard-Jones potential. The particle mesh Ewald (PME) method[13] was used to calculate the electrostatic interactions. Initially, the system was packed and energy minimized. Next, the system was cooled and heated by equilibrium simulations of 2 ns between (400 K, 1 bar) and (303 K, 1bar) for three cycles. This was followed by a short equilibrium simulation at 303 K and 1 bar for 10 ns. Finally, equilibrium system was performed for 800 ns.

$LiPF_6$ salt and EC/ EMC solvent are modeled based on the OPLS-AA (optimized potentials for liquid simulations with all atom model) force field.[14, 15] The Lennard-Jones potential and bonded interactions of all four species are obtained using LigParGen web server.[16] While the partial atomic charges for $LiPF_6$ are directly available from OPLS-AA force field and widely used, the partial charges of EC and EMC are separately calculated using RESP method as mentioned in the main text. First, partial atomic charges of EC and EMC were first calculated via density functional theory (DFT) method with B3LYP/6-31G* basis sets using Gaussian package.[17] Then, the partial atomic charges were fitted using the RESP method via the Antechamber package.[18] The partial charges for all atomic species used in simulations are summarized in Fig. S5.

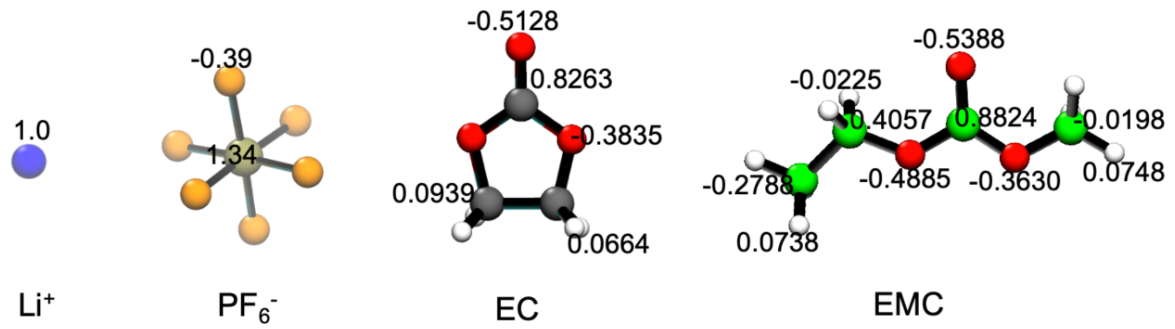

Figure S5. Charges (in unit of *e*) for all atomic species $LiPF_6$, EC, and EMC. Color codes for atoms: hydrogen in white, lithium in blue, carbon of EC in grey, carbon of EMC in green, oxygen in red, fluorine in yellow, and phosphorus in tan.

III. Obtaining transport coefficients

The transport coefficients are evaluated from the term equivalent to mean square displacement in Eqs. 3a and 3b in the main text. This term is defined as $\mathrm{MSD}_{ij}^{r}(t) = \left\langle \frac{1}{N_i}\sum_\alpha\left[\boldsymbol{r}_{i,\alpha}^{r}(t) - \boldsymbol{r}_{i,\alpha}^{r}(0)\right]\cdot\frac{1}{N_j}\sum_\beta\left[\boldsymbol{r}_{j,\beta}^{r}(t) - \boldsymbol{r}_{j,\beta}^{r}(0)\right]\right\rangle$, where r is the reference frame of either A or B. The transport coefficients are related to this term by $L_{ij}^{r} = \frac{V}{k_B T}\frac{1}{6}\lim_{t\to\infty}\frac{d}{dt}\mathrm{MSD}_{ij}^{r}(t)$. The window size for computing MSD_{ij}^{r} is 20 ns and the ensemble average accounts for all available time of origins of each simulation trajectory. Figure S6 shows $\mathrm{MSD}_{ij}^{r}(t)$s for the six transport coefficients under the reference frame of both A and B. The diffusive regime ($\mathrm{MSD}_{ij}^{r}\sim t$) is achieved within the time window. L_{ij}^{r}s are obtained by fitting the slope of $\mathrm{MSD}_{ij}^{r}(t)$ in the regime of 1-10ns, which is denoted as shaded blocks. The error bars are evaluated by first dividing the $\mathrm{MSD}_{ij}^{0}(t)$ data in the fitting regime into 10 consecutive blocks with the same size and then obtaining the standard error between slopes fitted in all data blocks.

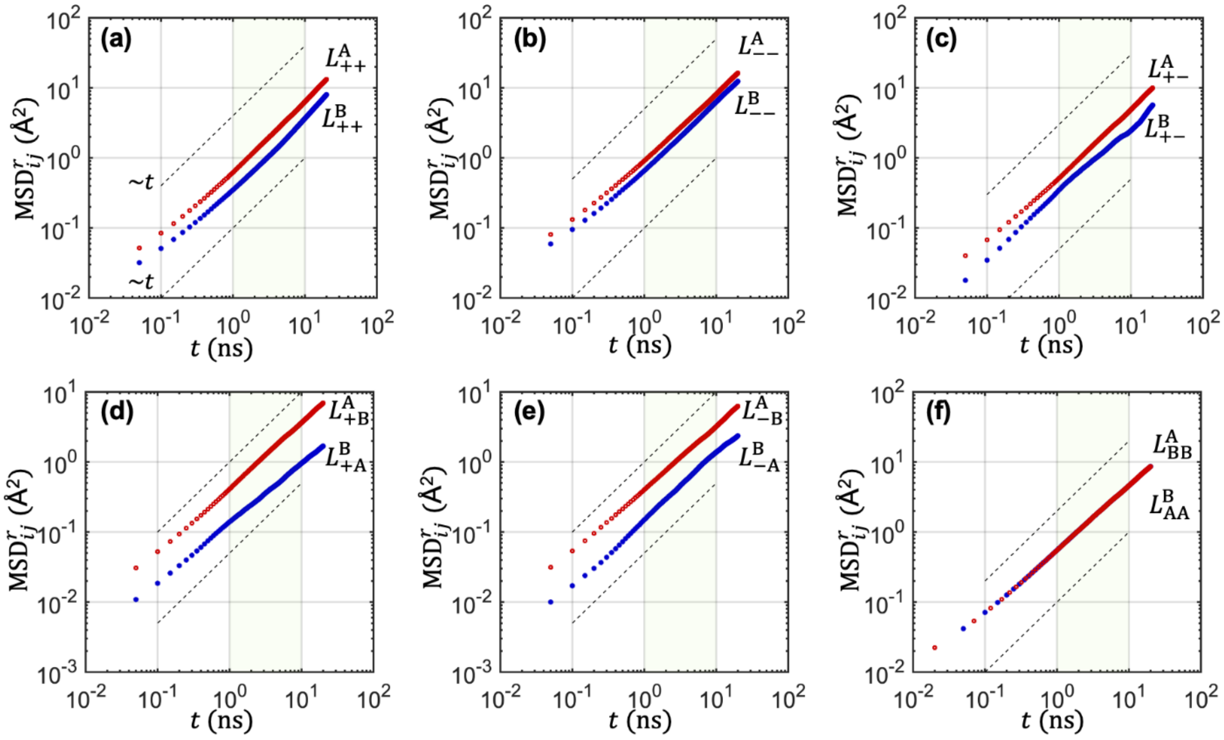

Figure S6. Mean square displacement term for each transport coefficient under a reference velocity of solvent A or B: MSD_{ij}^{r} for **(a)** cation-cation, **(b)** anion-anion, **(c)** cation-anion correlations, **(d)** cation-solvent, **(e)** anion-solvent, **(f)** solvent-solvent correlations. The dashed lines denote a diffusive regime where $\mathrm{MSD}_{ij}^{\mathrm{r}}(t)\sim t$.

IV. Determining the coordination number

The Li^+ coordination number is calculated based on radial distribution functions. Figure S7 shows the $\mathbf{g}(\boldsymbol{r})$ for carbonyl oxygen from EC, carbonyl oxygen from EMC, and phosphorus atom from PF_6^- around Li^+. The local of the valley outside of the primary peak is used as the cut-off distance to determine coordination number of EC, EMC, and PF_6^-.

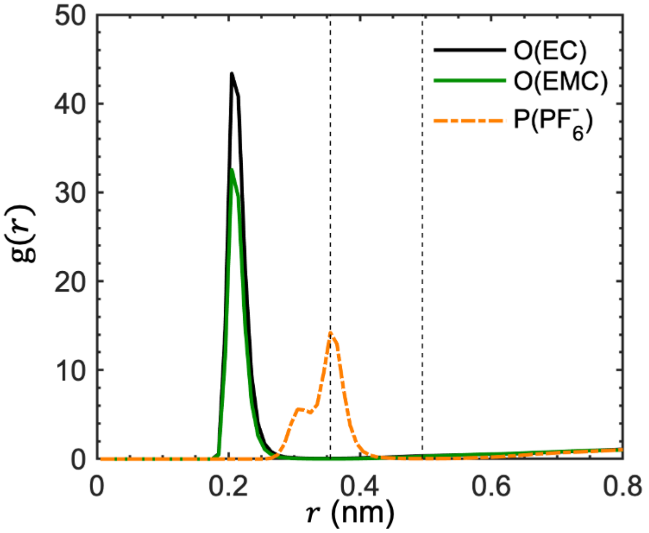

Figure S7. Radial distribution function of carbonyl oxygen from EC, carbonyl oxygen from EMC, and phosphorus atom from PF_6^- around Li^+. The two vertical dashed lines denote the valley outside of the primary peaks at r = 0.35 nm (for both Li-O(EC) and Li-O(EC)) and r = 0.50 nm (for Li-P(PF_6^-)), respectively.

To confirm the negligible anion solvation, we examine the radial distribution functions $g(r)$ for both the cation and anion with the two solvents, as shown in Fig. S8. The cation-solvent interactions are represented by $g(r)$ between Li^+ and carbonyl oxygens of EC and EMC; the anion-solvent interactions are represented by $g(r)$ between F in PF_6^- and hydrogen atoms in EC and EMC. Note that only hydrogen atoms at the two ends of EMC are considered due to their positive atomic charges. It can be clearly seen that $g(r)$ between the anion and the solvents is almost featureless, in stark contrast to the strong peaks in the case of the cation. This demonstrates a much weaker anion-solvent interaction than cation-solvent interaction, indicating that the anion solvation can be safely neglected in our analysis.

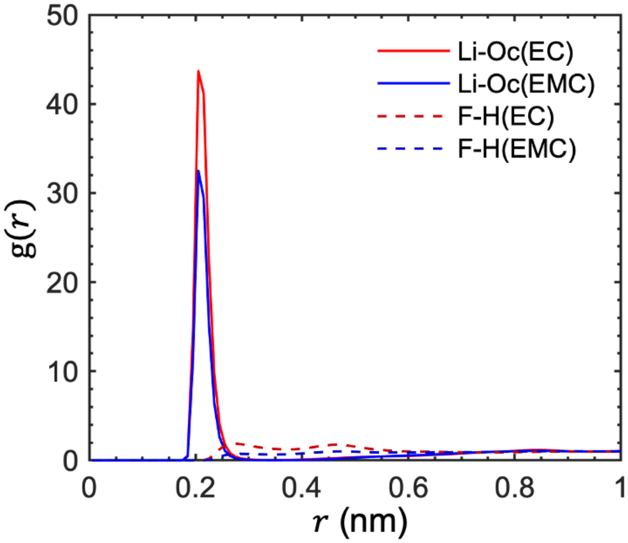

Figure S8. Radial distribution functions of Li^+ to carbonyl oxygen atoms (Oc) of EC and EMC, and F (PF_6^-) to hydrogen atoms (H) of EC and EMC.

V. Cluster approximation

The velocity of cluster type k carrying a net charge of q_k under an electric-field E is $q_k E D_k / k_B T$ based on the Einstein relation, where D_k is the self-diffusion coefficient. Because the composition of a cluster type k is not fixed, different D_k can exist for the same cluster type. Considering this variability, the velocity of cluster type k with composition l is denoted as $v_k^l = q_k E D_k^l / k_B T$. The velocity of species r in cluster type k is averaged through all possible compositions as $v_{r,k} = \sum_l f_{r,k}^l v_k^l \, / \sum_l f_{r,k}^l$, where $f_{r,k}^l$ is the fraction of species r in cluster type k with composition l. These velocities are normalized by the absolute velocity of anion under the same electric-field, which is equal to $v_{nor} = eED_{anion}/k_B T$. The normalized $v_{r,k}$ thus becomes $\sum_l f_{r,k}^l \frac{q_k}{e} \frac{d_{anion}}{d_k^l} \, / \sum_l f_{r,k}^l$ based on Stokes-Einstein equation, where d_k^l is the effective diameter of cluster type k with composition l. d_{anion}/d_k^l is then estimated by $\left(M_{anion}/M_k^l\right)^{1/3}$, where M is the molar mass. The contribution of velocities in each cluster type to species velocities $\bar{v}_r$ is given by $f_{r,k} v_{r,k}$, where $f_{r,k}$ is the fraction of species r in cluster type k as shown in Fig. S9.

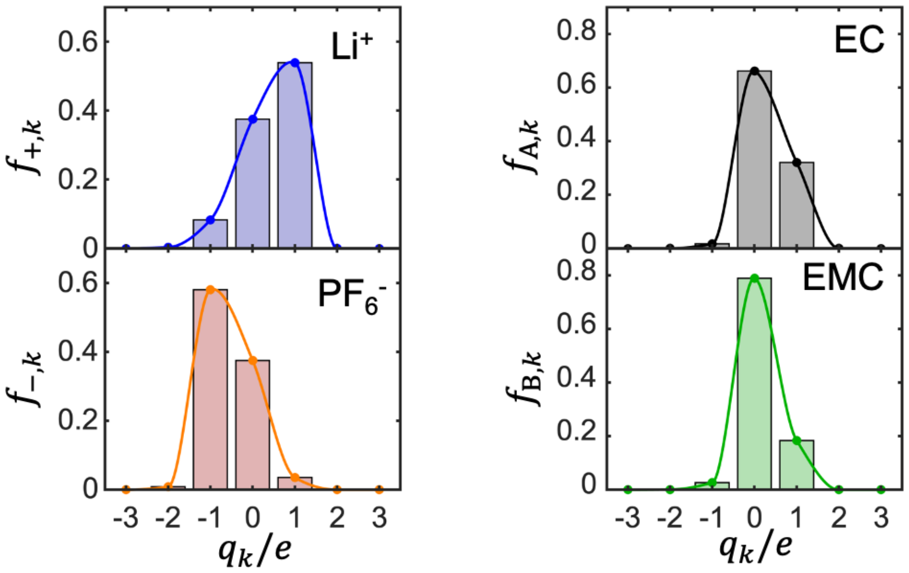

Figure S9. Fraction of cations, anions, EC and EMC within clusters carrying different net charge q_k.

References

1. Fang, Y.; Yushmanov, P. V.; Furó, I., Improved accuracy and precision in electrophoretic NMR experiments. Current control and sample cell design. *J. Magn. Reson.* **2020,** *318*, 106796.
2. Halat, D. M.; Fang, C.; Hickson, D.; Mistry, A.; Reimer, J. A.; Balsara, N. P.; Wang, R., Electric-field-induced spatially dynamic heterogeneity of solvent motion and cation transference in electrolytes. *Phys. Rev. Lett.* **2022,** *128* (19), 198002.
3. Jerschow, A.; Müller, N., Convection compensation in gradient enhanced nuclear magnetic resonance spectroscopy. *J. Magn. Reson.* **1998,** *132* (1), 13-18.
4. He, Q.; Wei, Z., Convection compensated electrophoretic NMR. *J. Magn. Reson.* **2001,** *150* (2), 126-131.
5. Karatrantos, A.; Khan, M. S.; Yan, C.; Dieden, R.; Urita, K.; Ohba, T.; Cai, Q., Ion Transport in Organic Electrolyte Solutions for Lithium-ion Batteries and Beyond. *J. Energy Power Technol.* **2021,** *3* (3), 1-1.
6. Zhang, Z.; Madsen, L. A., Observation of separate cation and anion electrophoretic mobilities in pure ionic liquids. *J. Chem. Phys.* **2014,** *140* (8), 084204.
7. Halat, D. M.; Mistry, A.; Hickson, D.; Srinivasan, V.; Balsara, N. P.; Reimer, J. A., Transference Number of Electrolytes from the Velocity of a Single Species Measured by Electrophoretic NMR. *J. Electrochem. Soc.* **2023**.
8. Wang, A. A.; Greenbank, S.; Li, G.; Howey, D. A.; Monroe, C. W., Current-driven solvent segregation in lithium-ion electrolytes. *Cell Reports Physical Science* **2022,** *3* (9), 101047.
9. Abraham, M. J.; Murtola, T.; Schulz, R.; Páll, S.; Smith, J. C.; Hess, B.; Lindahl, E., GROMACS: High performance molecular simulations through multi-level parallelism from laptops to supercomputers. *SoftwareX* **2015,** *1*, 19-25.
10. Hess, B.; Bekker, H.; Berendsen, H. J.; Fraaije, J. G., LINCS: a linear constraint solver for molecular simulations. *J. Comput. Chem.* **1997,** *18* (12), 1463-1472.
11. Bussi, G.; Donadio, D.; Parrinello, M., Canonical sampling through velocity rescaling. *J. Chem. Phys.* **2007,** *126* (1), 014101.
12. Berendsen, H. J.; Postma, J. v.; Van Gunsteren, W. F.; DiNola, A.; Haak, J. R., Molecular dynamics with coupling to an external bath. *J. Chem. Phys.* **1984,** *81* (8), 3684-3690.
13. Darden, T.; York, D.; Pedersen, L., Particle mesh Ewald: An N· log (N) method for Ewald sums in large systems. *J. Chem. Phys.* **1993,** *98* (12), 10089-10092.
14. Jorgensen, W. L.; Maxwell, D. S.; Tirado-Rives, J., Development and testing of the OPLS all-atom force field on conformational energetics and properties of organic liquids. *J. Am. Chem. Soc.* **1996,** *118* (45), 11225-11236.
15. Sambasivarao, S. V.; Acevedo, O., Development of OPLS-AA force field parameters for 68 unique ionic liquids. *J. Chem. Theory Comput.* **2009,** *5* (4), 1038-1050.
16. Dodda, L. S.; Cabeza de Vaca, I.; Tirado-Rives, J.; Jorgensen, W. L., LigParGen web server: an automatic OPLS-AA parameter generator for organic ligands. *Nucleic acids research* **2017,** *45* (W1), W331-W336.
17. Frisch, M.; Trucks, G.; Schlegel, H.; Scuseria, G.; Robb, M.; Cheeseman, J.; Scalmani, G.; Barone, V.; Petersson, G.; Nakatsuji, H., Gaussian 16, Gaussian. *Inc., Wallingford CT* **2016,** *2016*.
18. Wang, J.; Wolf, R. M.; Caldwell, J. W.; Kollman, P. A.; Case, D. A., Junmei Wang, Romain M. Wolf, James W. Caldwell, Peter A. Kollman, and David A. Case," Development and testing of a general amber force field" Journal of Computational Chemistry (2004) 25 (9) 1157–1174. *J. Comput. Chem.* **2005,** *26* (1), 114-114.
